# Changes in the Population Size of Calbindin D-28k-Immunoreactive Enteric Neurons in the Porcine Caecum under the Influence of Bisphenol A: A Preliminary Study

**DOI:** 10.3390/toxics9010001

**Published:** 2020-12-28

**Authors:** Ignacy Gonkowski, Slawomir Gonkowski, Ewa Dzika, Joanna Wojtkiewicz

**Affiliations:** 1 Students’ Scientific Club of Pathophysiologists, Department of Human Physiology and Pathophysiology, School of Medicine, University of Warmia and Mazury, 10-082 Olsztyn, Poland; gon.ign@wp.pl; 2 Department of Clinical Physiology, Faculty of Veterinary Medicine, University of Warmia and Mazury in Olsztyn, Oczapowskiego Str. 13, 10-718 Olsztyn, Poland; 3 Department of Medical Biology, Faculty of Health Sciences, University of Warmia and Mazury in Olsztyn, Żołnierska 14C Str., 10-561 Olsztyn, Poland; e.dzika@uwm.edu.pl; 4 Department of Human Physiology and Pathophysiology, School of Medicine, University of Warmia and Mazury, 10-082 Olsztyn, Poland

**Keywords:** endocrine disruptors, enteric nervous system, calcium binding proteins, domestic pig, toxicology

## Abstract

Calbindin D-28k (CB) is a calcium-binding protein widely distributed in living organisms that may act as a calcium buffer and sensory protein. CB is present in the enteric nervous system (ENS) situated in the gastrointestinal tract, which controls the majority of activities of the stomach and intestine. The influence of various doses of bisphenol A (BPA)—a chemical compound widely used in plastics production—on the number and distribution of CB-positive enteric neuronal cells in the porcine caecum was investigated with an immunofluorescence technique. The obtained results showed that low dosages of BPA resulted in an increase in the number of CB-positive neuronal cells in the myenteric (MP) and inner submucous (ISP) plexuses, whereas it did not alter the number of such neuronal cells in the outer submucous plexus (OSP). High dosages of BPA caused the increase in the amount of CB-positive perikarya in all the above-mentioned kinds of the caecal neuronal plexuses. These observations strongly suggest that CB in the ENS participates in the processes connected with the toxic activity of BPA. Most likely, the changes noted in this experiment result from the adaptive and protective properties of CB.

## 1. Introduction

Calbindin D-28k (CB) is a calcium-binding protein belonging to the EF hand family proteins [1,2]. It is present in many internal organs and acts as a calcium buffer and sensory protein [3,4,5,6]. In the nervous system, CB first of all occurs in the brain, where it participates in the maintenance of calcium homeostasis and plays a crucial role in the neuroprotective processes [7,8]. Moreover, CB is present in the parasympathetic, sympathetic and sensory peripheral nervous systems [9,10,11,12]. It has also been found the enteric nervous system (ENS) [13].

**Figure 1:**
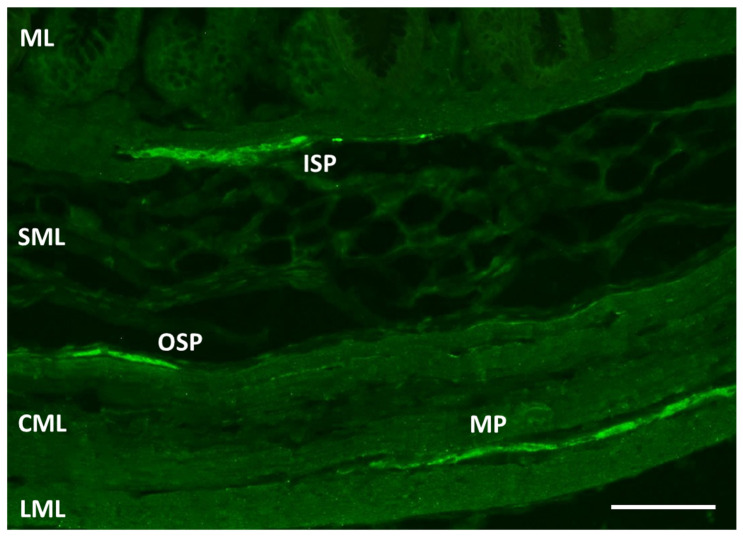
The enteric nervous system in the caecum of the domestic pig shown by labeling with pan-neuronal marker protein gene product 9.5 (PGP 9.5): ML: mucosal layer, ISP: inner submucous plexus, SML: submucosal layer, OSP: outer submucous plexus, CML: circular muscular layer, MP: myenteric plexus and LML: longitudinal muscular layer. Scale bar: 100 µm.

The ENS, characterized by complex anatomy, a considerable neurochemical diversity and autonomy against the central nervous system, is situated in the wall of the esophagus, stomach and intestines [14,15]. Its organization depends on the species [16,17]. In the intestines of domestic pigs, the ENS is composed of three kinds of plexuses [16,18,19,20]: (1) the myenteric plexus (MP) in the intestinal muscular layer, (2) the outer submucous plexus (OSP) in the submucosal layer in the vicinity of the muscular layer and (3) the inner submucous plexus (ISP), also in the submucosal layer but close to the mucosal layer (Figure 1).

CB occurring in the ENS of various mammal species [13,21,22,23,24,25] is involved in the sensory stimuli conductions and regulations of calcium homeostasis, and its exact roles probably depend on the species [13]. In the guinea pig, CB is mainly distributed in the intrinsic primary sensory neurons (IPANs) [16,26,27]. Since the processes of CB-positive IPANs form varicose structures around other types of the enteric neurons, CB not only participates in sensory conduction but, also, may affect other intestinal functions (e.g., intestinal motility, blood flow and secretory activity) [28,29]. In other species, CB was found in different types of neurochemically diversified enteric neuronal cells, and its exact functions are not fully clear [13,30,31].

The ENS changes under physiological and pathological factors, such as, for example, diet, maturation, inflammation, metabolic disorders and toxic substances in the food [18,19,23,32,33,34]. These factors first of all affect the expression of active substances by the enteric neuronal cells but, also, change their number and appearance, as well as functional and electrophysiological characteristics [18,19].

On the other hand, bisphenol A (BPA), a chemical compound commonly utilized in the plastics industry and included in various everyday objects [35], shows adverse effects on various internal organs [35,36,37,38]. However, since BPA gets into the organism mainly with food, the gastrointestinal tract and ENS are particularly exposed to its harmful effects [19]. Previous investigations have shown that BPA induces changes in the expression of various neurochemical factors (e.g., galanin, vasoactive intestinal polypeptide, nitric oxide and acetylcholine in the ENS of the stomach and small intestine [19,39,40,41], but the knowledge about the influence of this substance on the ENS in the large intestine is extremely scanty and limited to nitric oxide synthase and neuregulin 1 [41,42].

Admittedly the absorption of BPA takes place in the initial part of the jejunum, where (in enterocytes) it undergoes glucuronidation [43], but the caecum plays important roles in its transformation. It is because the majority of BPA-glucuronide synthetized in jejunal enterocytes is transported back into the intestinal lumen and, in the large intestine (under the impact caecum microbiota), undergoes the renewed deconjugation to free BPA [43]. Therefore, the wall of the caecum and colon is subjected to the impact of BPA to a large extent.

The domestic pig was selected for this experiment because of significant similarities in the structures of the human and porcine nervous systems (especially the ENS); which causes the pig to seem to be a good model species for studies on mechanisms appearing in the human organism under the impact of pathological factors [44].

This study aimed to determine the changes in the CB-immunoreactive enteric neuronal cells in the porcine caecum induced by BPA administered in various doses. The obtained results, on the one hand, will allow the expanding of information about the influence of BPA on mammal large intestines, and on the other hand, will answer the question if CB-positive enteric nervous structures participate in processes occurring during the response of the body to BPA impact.

## 2. Materials and Methods

### 2.1. Experimental Animals and BPA Dosing

Fifteen eight-week-old sows of the Pitrain × Duroc breed were included in this study. All experimental actions were performed in accordance with ethic approval issued by the Local Ethical Committee for Experiments on Animals in Olsztyn (Poland) (decision numbers 28/2013 of 22 May 2013 and 65/2013/DLZ of 27 November 2013). Pigs were randomly divided into three groups of five individuals. Each group was kept in a separate pen dedicated to juvenile pigs. All pigs were fed twice a day with the same commercial feed and had access to water ad libitum.

The plan of the experiment was previously described by Szymanska et al. [19]. In brief, the control pigs (C Group) were orally treated with gelatin capsules during the morning feeding. Animals from Group I received identical capsules filled with BPA (catalog no. 239658, Sigma Aldrich, Poznan, Poland) in an amount 0.05 mg/kg body weight (b.w.)/day, and pigs from Group II were treated with a higher amount of BPA (0.5 mg/kg b.w./day). All groups of pigs were treated with capsules for 28 days. Then, the animals were euthanized through premedication with Stressnil (Janssen, Belgium; 75 μL/kg of b.w.) and the administration of an overdose of sodium thiopental (Thiopental, Sandoz, Kundl, Austria).

### 2.2. Collection of the Fragments of the Caecum and Preparation to Immunofluorescence Method

Promptly after euthanasia, the fragments of the caecum (of approximately 3 cm in length) were collected from the same place located 10 cm from the ileocecal valve. Immediately after the sample collection, the fragments of the caecum were fixed in 4% buffered paraformaldehyde (pH 7.4). The fixation was performed in room temperature (rt) and lasted 1 h. Then, tissues were washed in phosphate buffer for 3 days and stored in 18% phosphate-buffered sucrose. Fragments of the caecum were at 5 °C. The next stages of tissue preparation (performed at least after three weeks of storage) involved: freezing of the intestinal fragments at −22 °C, cutting them into 14-μm-thick sections with the cryostat (HM 525, Microm International, Dreieich, Germany) and mounting of the intestinal slices on microscopic slides. The fragments of the caecum on slices were put into −20 °C until labeling.

### 2.3. Immunohistochemistry Method

To investigate the number of CB-positive enteric neuronal cells, the typical double-labeling immunofluorescence method, utilized previously by Palus et al. [34], was used. This method included the following steps (performed at rt) separated from each other by a rinsing in PBS (3 × 15 min.): (1) drying of the slides with intestinal fragments for 1 h; (2) incubation of intestinal fragments in a solution containing 10% normal goat serum, 0.1% bovine serum albumin, 0.01% NaN3, Triton X-100 and thimerosal in PBS for 45 min; (3) overnight incubation with the mixture of mouse antibody against protein gene product 9.5 (PGP 9.5, applied as a pan-neuronal marker obtained from Bio-Rad, Hercules, CA, USA (code no. 7863-2004, working dilution 1:1000) and rabbit antibody against CB obtained from Swant, Marly, Switzerland (code no. CB-38, working dilution 1:4000); (4) a one-hour incubation with the mixture of species-specific antibodies conjugated with the appropriate fluorochromes: Alexa Fluor 488-conjugated donkey anti-mouse IgG (H + L) and Alexa Fluor 546-conjugated goat anti-rabbit IgG (H + L) (both obtained from Thermo Fisher Scientific, Waltham, MA, USA; working dilution 1:1000) and (5) the application of buffered glycerol on the fragments of the caecum and covering with cover slips. To verify the labeling specificity, routine tests (such as pre-absorption, omission and replacement tests) of using antibodies were made up.

### 2.4. Counting of CB-Positive Neurons

Labeled slices of the caecum were studied using an Olympus BX51 epi-fluorescence microscope with appropriate filter (Olympus, Tokyo, Japan). The determination of the percentage of neuronal cells containing CB consisted in the evaluation of at least 500 cells immunoreactive to PGP 9.5 located in each kind of the enteric plexus for the occurrence of CB, and the number of cell bodies containing PGP 9.5 included into the experiment was regarded as 100%. To avoid the dual counting of the same cell bodies, the slices of the caecum on which the counting of cells was performed were distant from each other by at least 200 µm.

### 2.5. Evaluation of the Impact of BPA on the Total Population of Enteric Neuronal Cells

To study the eventual impact of BPA administration on the entire population of the neuronal cells in the caecal ENS, the number of all cell bodies containing PGP 9.5 in each kind of intramural ganglia were evaluated in ten fragments of the caecum derived from each pig (distant from each other by at least 400 µm).

### 2.6. Statistical Analysis

The results were pooled and presented as the mean ± SEM. During this experiment, the univariate ANOVA test (GraphPad Prism v. 3.0, GraphPad Software Inc., San Diego, CA, USA) was used to perform the statistical analysis. The differences were regarded as statistically significant at *p* ≤ 0.05.

## 3. Results

**Figure 2:**
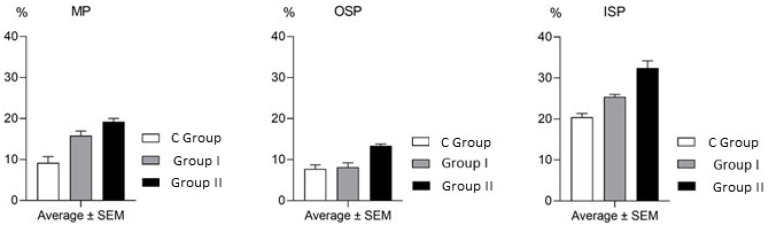
Visualization of the impact of bisphenol A (BPA) on the percentage of calbindin D-28k-immunoreactive enteric neuronal cells in the myenteric (MP), outer submucous (OSP) and inner submucous (ISP) plexuses in the porcine caecum.

**Figure 3:**
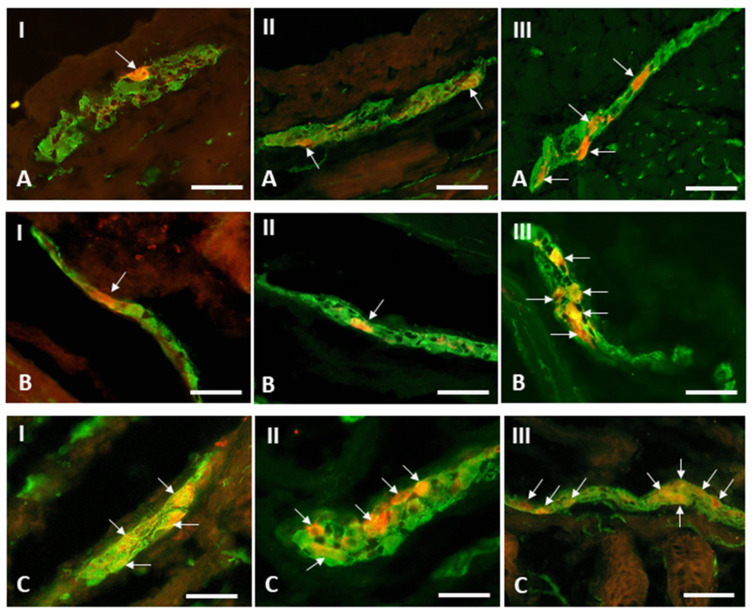
Myenteric plexus (**A**), outer submucous plexus (**B**) and inner submucous plexus (**C**) in the porcine caecum in control animals (**I**) and animals treated with low (**II**) and high (**III**) doses of bisphenol A, labeled against pan-neuronal marker PGP 9.5 (green) and calbindin (red). Calbindin-positive neurons are indicated with arrows. Scale bar: 50 µm.

In this experiment, the occurrence of CB-immunoreactive cells was noted in all kinds of the enteric plexuses located in the porcine caecum. Such cells were noted both in pigs under physiological conditions and animals exposed to BPA (Table 1 and Figure 2 and Figure 3).

Under physiological conditions, the percentage of neurons containing CB located in the MP and OSP achieved 9.18% ± 1.53% and 7.75% ± 0.96% of all cells immunoreactive to PGP 9.5, respectively. In turn, the percentage of neurons immunopositive to CB in the ISP was significantly higher and was at the level of 20.42% ± 0.92% of all neurons immunoreactive to PGP 9.5 (Table 1).

**Figure 4:**
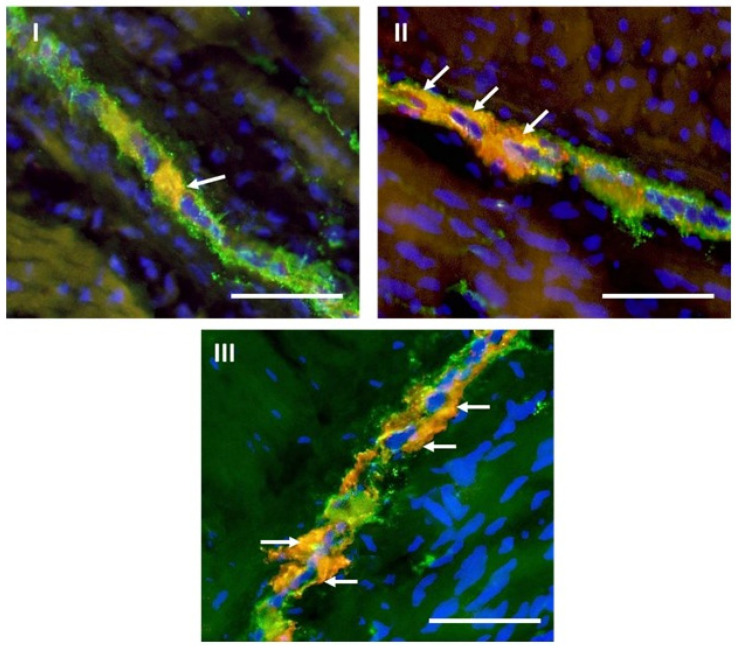
Myenteric plexus in the porcine caecum in control animals (**I**) and animals treated with low (**II**) and high (**III**) doses of bisphenol A, labeled against pan-neuronal marker PGP 9.5 (green), calbindin (red) and a marker of cell nuclei-4′,6-diamidino-2-phenylindole (DAPI) (blue). Calbindin-positive neurons are indicated with arrows. Scale bar: 50 µm.

The impact of BPA on the population of CB-immunoreactive enteric neuronal cells in the caecum was investigated during this experiment. In the MP (Figure 4), the administration of both dosages of BPA resulted in the increase in the percentage of enteric neuronal cells containing CB. In pigs receiving low dosages of BPA, the percentage of neuronal cells immunopositive to CB achieved 15.80% ± 1.14% of all cells containing PGP 9.5. In the case of pigs in which high dosages of BPA were administered, this increase was even more pronounced, because the number of CB-positive cells was at the level of 19.23% ± 0.77% of the total population of PGP 9.5–positive cell bodies (Table 1 and Figure 2).

Differently from the MP, low dosages of BPA did not affect the population size of the CB-positive neurons in the OSP (Figure 2 and Figure 3). The percentage of such neuronal cells in pigs after the administration of low dosages of BPA amounted to 8.15% ± 1.07% of the total population of cells containing PGP 9.5, and this value did not show statistically significant differences in comparison with animals not treated with BPA (Table 1). In turn, in pigs receiving high dosages of BPA, the percentage of neuronal cells immunoreactive to CB in the OSP increased to 13.42% ± 0.40% of the total number of PGP 9.5–immunoreactive cell bodies and was clearly higher than in the pigs of the C Group (Table 1 and Figure 2).

In the ISP, both dosages of BPA used in the experiment resulted in the increase in the percentage of neuronal cells containing CB (Figure 3C). In animals in which low dosages of BPA were administered, the amount of such neuronal cell bodies increased to 25.40% ± 0.59% of the total number of PGP 9.5-immunoreactive cells, and the administration of high dosages of BPA caused this value to be even higher and achieved a result of 32.43% ± 1.74% (Table 1 and Figure 2).

**Figure 5:**
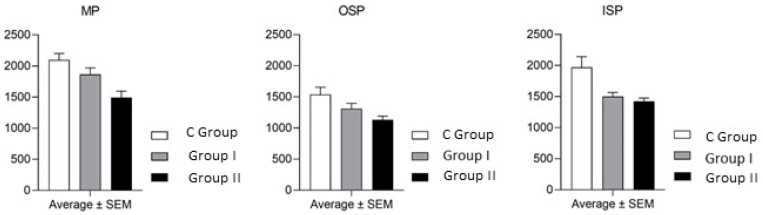
Visualization of the impact of BPA on the total population of the enteric neuronal cells (immunoreactive to PGP 9.5) in the myenteric (MP), outer submucous (OSP) and inner submucous (ISP) plexuses in the porcine caecum.

During the present study, the impact of BPA on the entire population of PGP 9.5-immunoreactive enteric neurons cells was confirmed. Generally, BPA caused a decrease in the number of the enteric neuronal cell bodies containing PGP 9.5, but the intensity of these fluctuations was conditional upon the dosage of BPA and kind of the enteric plexus (Table 2 and Figure 5).

As regards low dosages of BPA, a statistically significant decrease in the number of cell bodies immunoreactive to PGP 9.5 was confirmed only in the ISP, where this value decreased from 1969 ± 174.0 to 1501 ± 65.34. In the MP and OSP, the number of the total enteric neuronal cell bodies was also lower, but there were no statistically significant differences between animals of the C Group and pigs receiving low dosages of BPA (Table 2 and Figure 5).

After the administration of high dosages of BPA, the decrease in the population size of the cells immunoreactive to PGP 9.5 was better visible. The number of the total neuronal cells increased to 1493 ± 101.5, 1130 ± 59.95 and 1422 ± 53.22 in the MP, OSP and ISP, respectively, and these amounts were statistically significantly lower in comparison to the control animals (Table 2 and Figure 5).

## 4. Discussion

In the present study, CB-positive neuronal cells were found in all three kinds of the intramural nervous plexuses in the caecum, which confirms that CB plays important and multidirectional roles in the ENS. These findings correspond to previous investigations conducted on other mammal species [13,21]. In turn, the comparison of previous observations on other mammal species [13,21,30,31] with the results of the present experiment shows clear interspecies differences in the distribution of CB in the ENS. Moreover, previous findings conducted on other segments of the porcine gastrointestinal tract [23] differ from the observations made during the present study. This fact shows that the number of CB-positive enteric neurons explicitly depends on the intestinal fragment and suggests that the exact functions of this substance are probably also different in particular parts of the digestive tract. In turn, the presence of CB-immunoreactive perikarya in all kinds of the intramural nervous plexuses suggests that CB is present in different types of neurons within the ENS and in the domestic pig (contrary to the guinea pig [26,27,28,29] and similarly to other mammal species [13,31]) and cannot be considered as a marker typical for IPANs. The exact determination of the enteric neuronal populations in the porcine ENS in which CB may occur, and the exact functions of this substance, require further studies.

During this experiment, the impact of BPA administration on the population of CB-immunoreactive neurons in the caecum was noted. Previous studies on the influence of BPA on the population of cells containing CB concerned only the central nervous system in rodents and showed that BPA, depending on the following: dose, the manner of administration, part of the brain and animal species, does not affect the number of CB-positive neurons or may cause an increase in the population of such neurons [45,46].

Generally, during the present study, it was shown that the treatment with BPA resulted in an increase in the number of perikarya containing CB, but the intensity of the changes depended on the dose of BPA and type of the enteric plexus. Interestingly, even the administration of low dosages of BPA resulted in an increase in the number of CB-immunoreactive perikarya in the MP and ISP. It must be stressed that the dosage of BPA at the level of 0.05 mg/kg b.w./day is recommended in some countries as a tolerable daily intake and regarded as safe for humans and animals [47]. However, the observations performed during the present experiment showed that such a dosage of BPA can influence the neurochemical profile of neuronal cells in the ENS and, therefore, is not neutral for humans and animals. It is in agreement with previous findings, in which descriptions of the impact of such doses of BPA on the nervous system in the liver, uterus and other segments of the gastrointestinal tract were noted [19,48,49].

The fluctuations in the population size of CB-positive enteric neuronal cells observed in this experiment are one of responses of the ENS (apart from fluctuations in the levels of other active substances noted in previous studies [19,39,40,41]) to the neurotoxic activity of BPA. The increase of the percentage of caecal intramural neurons containing CB observed in this investigation may partially result from the reduction of the abundance of the total population of neuronal cells in the ENS under the impact of BPA. Such BPA-induced reduction in the population of neuronal cells was noted not only in the present investigation but, also, the previous experiments conducted on the brain [50]. However, the degree of reduction of the total number of the enteric neuronal cells is relatively small (smaller than the increase of the population of CB-immunoreactive neurons, especially in pigs after the administration of low dosages of BPA), which suggests that the observed changes result from the synthesis of CB in neurons, which, under physiological conditions, do not produce this substance.

These changes might be the manifestation of protective and adaptive reactions. Previous studies, which mainly concerned the central nervous system, showed that BPA inhibits synaptogenesis [51], distorts ions transport and the production of neuroproteins [52], negatively affects the growth and development of dendrites and axons [53,54] and induces oxidative stress reactions in neuronal cells [55]. Moreover, it is known that BPA influences the sensory stimuli conduction [56], may affect the higher nervous activity [57] and some previous studies described connections between an exposure to BPA and the frequency of neurodegenerative diseases [58].

Information concerning the impact of BPA on the peripheral nervous system, contrary to the brain, is much more limited, but previous studies have reported that BPA may affect the sodium transport in sensory neurons and change the expression of active substances in the neurons and nerve fibers innervating the internal organs [19,48,49,59]. It has also been reported that BPA affects the neurochemical characterization of the ENS in the small intestine, stomach and descending colon. Namely, BPA changes the number of the enteric neuronal cells containing various neurochemical substances, such as acetylcholine, vasoactive intestinal polypeptide, substance P, nitric oxide, galanin and cocaine and amphetamine-regulated transcript peptide (CART peptide) [19,39,40,41]. The characteristics and severity of the changes observed within the ENS after the administration of BPA are determined by the segment of the digestive tract, type of the enteric plexuses, dosage of BPA and neurochemical factor studied. Generally, in the small intestine, BPA causes the reduction of the population of the enteric cholinergic perikarya and an increase in the number of neuronal cells immunoreactive to the substance P, galanin and the CART peptide [19,39,40]. In turn, the impact of BPA on the enteric neuronal cell bodies containing nitric oxide and vasoactive intestinal polypeptide is inconclusive. In some segments of the digestive tract, the administration of BPA resulted in the decrease in the number of such perikarya; in other segments, the increase of their populations [19,39,40,41]. However, the detailed mechanisms connected with the impact of BPA in the ENS are not clear.

In turn, the main reason for the changes observed in this experiment may result from the influence of BPA on the cellular calcium metabolism. It is known that one of the ways by which BPA affects the cells is disorder of the calcium ion transport and, hence, disturbances in calcium signaling [60,61]. Previous investigations regarding the impact of BPA on calcium metabolism in the nervous tissues are rather scant [61,62,63,64]. Moreover, the results concerning this issue are not explicit, and the characteristics of the influence of BPA on the calcium channels depend on the doses of BPA, the part of the nervous system and the experimental model. Some studies reported that BPA interacts with extracellular parts of voltage-activated Ca^2+^ channels and inhibits their activity [62]. In turn, other studies described the BPA-induced activation of calcium ion channels and enhanced influx of Ca^2+^ ions into neuronal cells [61,63,64]. Higher levels of Ca^2+^ inside the neurons, observed after the administration of BPA [61,65], leads to the exacerbation of apoptosis, changes in the mitochondria, oxidative stress reactions and neurodegenerative processes [63,66].

In order to prevent these reactions, neurons start to synthetize CB, because it is a substance that activates Ca^2+^-ATP enzymes and plays a crucial role in the reduction of intracellular calcium levels and the protection of cells against excitotoxic damage caused by excessive amounts of calcium ions entering the cell body [67,68]. These protective roles make CB-rich neurons more resistant to damaging factors [7,69,70]. It is also known that various pathological processes often induce an increase in the synthesis of CB in neuronal cells [70,71,72]. Admittedly, the majority of the earlier investigations regarding this issue were performed on the central nervous system, but it can be expected that the fluctuations noted in this experiment resulted from similar neuroprotective and adaptive processes in the ENS in response to the BPA impact. A similar increase of the population of CB-immunoreactive neuronal cells, caused by protective reactions, was previously observed in the ENS in rats subjected to experimental diabetes [67], in which calcium dyshomeostatsis (similarly to BPA activity) also appeared [73].

One cannot exclude the fact that fluctuations observed in this experiment resulted from the direct neurotoxic impact of BPA and from other mechanisms associated with the multidirectional effects of this endocrine disruptor on the organism. One such activity may be the impact of BPA on the intestinal muscular cells. Experiments ex vivo showed that BPA clearly reduces the motor activity of duodenum through signaling pathways related to nitric oxide-mediated soluble guanylyl cyclase and, therefore, the activation of enteric inhibitory nitrergic moto neurons [74]. On the other hand, it is known that CB-positive varicose baskets of nerves, being the processes of IPANs, regulate the activity of myenteric interneurons and excitatory motoneurons [26,29]. Therefore, it can be assumed that the increase of the population size of CB-immunoreactive neuronal cells observed in this experiment may be associated with the compensatory stimulation of excitatory motoneurons, being an answer to the relaxant activity of BPA aimed at intestinal homeostasis maintenance.

In turn, taking into account the involvement of CB in the conductivity of sensory and pain stimuli within the ENS [25,28], the changes may be caused by the proinflammatory properties of BPA [75]. However, this thesis is unlikely, due to the fact that the dosages of BPA investigated in the present study were quite low and did not result in any pain signs in the experimental animals.

The obtained results confirm that CB in the ENS takes part in reactions to pathological factors. Until now, only a few investigations described that the population size of CB-immunoreactive enteric neurons may change under physiological (growth and maturation) and pathological (e.g., intestinal ischemia ulcerative colitis or viral infection) factors [25,76,77]. However, it should be pointed out that, until now, the physiological consequences of the fluctuations of the expression of CB in the ENS were unknown. Similar to the brain, where the functions of CB are better understood, it can be supposed that fluctuations observed in this investigation may result in the suppression of apoptosis and the death of neurons, assuring the correct functioning of synapses and increase of neuronal excitability [7,78,79,80].

## 5. Conclusions

The obtained results showed that BPA affects the population of CB-immunoreactive enteric neurons in the porcine caecum. Modifications of the number of neurons containing CB were noted even in the case of low dosages of BPA, which, in some countries, are regarded as safe for humans and animals. These observations indicate that BPA (also in low dosages) is not neutral for the organism and influences on the ENS in the large intestine. Moreover, the present results show that CB-positive takes part in reactions connected with the BPA impact. The increase in the number of the population size of neuronal cells containing CB may arise from both the decrease in the total amount of neuronal cells in the ENS under the impact of BPA, as well as from the synthesis of CB in neurons, which, under physiological conditions, do not produce this substance. This synthesis is probably associated with the neurotoxic properties of BPA and has the capacity to disturb the calcium homeostasis in neuronal cells. The changes observed in this investigation are probably linked to neuroprotection and adaptive reactions in response to the impact of BPA, but the exact explanation of the mechanisms responsible for the observed changes require further investigation.

## Figures and Tables

**Table 1:** The average percentage of calbindin (CB)–immunoreactive neuronal cells in the myenteric plexus (MP), outer submucous plexus (OSP) and inner submucous plexus (ISP) in the caecum of control pigs (C Group) and in pigs after administration of low (Group I) and high (Group II) dosages of bisphenol A. ENS: enteric nervous system.

Part of the ENS	C Group		Group I		Group II	
	A	B	A	B	A	B
MP						
Animal 1	526/75	14.26%	507/92	18.15%	582/128	21.99%
Animal 2	532/38	7.14%	519/61	11.75%	500/96	19.20%
Animal 3	507/35	6.90%	504/79	15.67%	507/94	18.54%
Animal 4	582/65	11.16%	518/81	15.63%	513/98	19.10%
Animal 5	557/36	6.46%	523/93	17.78%	526/91	17.30%
Average ± SEM	9.18 ± 1.53%		15.80 ± 1.14% * (*p* = 0.0084)		19.23 ± 0.77% * (*p* = 0.0004)	
OSP						
Animal 1	513/29	5.65	526/35	6.65	521/72	13.82
Animal 2	506/52	10.28	501/56	11.18	506/61	12.06
Animal 3	523/31	5.93	535/29	5.42	509/71	13.95
Animal 4	502/49	9.76	519/52	10.02	516/67	12.98
Animal 5	518/37	7.14	508/38	7.48	532/76	12.29
Average ± SEM	7.75 ± 0.96%		8.15 ± 1.07% (*p* = 0.7890)		13.42 ± 0.40% * (*p* = 0.0010)	
ISP						
Animal 1	526/117	20.81	509/129	25.34	538/179	33.27
Animal 2	543/103	18.97	523/121	23.14	506/176	34.78
Animal 3	518/92	17.76	514/135	26.26	523/194	37.09
Animal 4	523/119	22.75	502/132	26.29	520/153	29.42
Animal 5	536/117	21.83	524/136	25.95	536/148	27.61
Average ± SEM	20.42 ± 0.92%		25.40 ± 0.59% * (*p* = 0.018)		32.43 ± 1.74% * (*p* = 0.0003)	

A: The number of cells protein gene product (PGP) 9.5+/CB+ counted in particular animals. B: The percentage of CB-positive cells in relation to the number of PGP 9.5-positive cells (treated as 100%). Statistically significant differences (*p* ≤ 0.05) between the C Group and Group I, as well as between the C Group and Group II in particular types of the enteric plexuses are marked with *.

**Table 2:** The average total population of neuronal cells containing PGP 9.5 (counted on ten slices of the intestine) in the myenteric plexus (MP), outer submucous plexus (OSP) and inner submucous plexus (ISP) in the caecum of the control pigs (C Group) and in pigs after the administration of low (Group I) and high (Group II) dosages of bisphenol A.

Part of the ENS	C Group	Group I	Group II
MP			
Animal 1	1908	2043	1634
Animal 2	2198	1865	1412
Animal 3	2326	2108	1134
Animal 4	2260	1798	1698
Animal 5	1795	1516	1585
Average ± SEM	2097 ± 104.0	1866 ± 104.2 (*p* = 0.1545)	1493 ± 101.5 * (*p* = 0.0031)
OSP			
Animal 1	1323	1287	878
Animal 2	1562	1623	1004
Animal 3	1317	1229	1172
Animal 4	1962	1098	997
Animal 5	1524	1320	800
Average ± SEM	1538 ± 117.4	1311 ± 86.82 (*p* = 0.1596)	1130 ± 59.95% * (*p* = 0.0028)
ISP			
Animal 1	2318	1476	1297
Animal 2	1721	1697	1583
Animal 3	1415	1318	1321
Animal 4	2276	1426	1419
Animal 5	2115	1586	1492
Average ± SEM	1969 ± 174.0	1501 ± 65.34 * (*p* = 0.0358)	1422 ± 53.22 * (*p* = 0.0170)

Statistically significant differences (*p* ≤ 0.05) between the C Group and Group I, as well as between the C Group and Group II in particular types of the enteric plexuses are marked with *.

## Data Availability

All data presented in this study are available in this article.
